# Supplementary material for: Genome Mining of Pseudanabaena galeata CCNP1313 Indicates a New Scope in the Search for Antiproliferative and Antiviral Agents
Source: Microorganisms. 2024 Aug 9;12(8):1628. doi: 10.3390/microorganisms12081628 (PMC11356792; doi:10.3390/microorganisms12081628)
Supplement: Supplementary file 1 [file microorganisms-12-01628-s001.zip › microorganisms-3107126-supplementary.pdf]

Supplementary Table S1. The statistics of the *Pseudanabaena galeata* CCNP1313 genomic analysis

**Parameter**	**Value/characteristics**
Assembly Method	Flye v. 2.9
Assembly Name	*Pseudanabaena galeata* CCNP1313
Genome Representation	Full
Genome Coverage	456.0x
Sequencing Technology	Illumina NovaSeq; Oxford Nanopore GridION
Annotation Method	Best-placed reference protein set; GeneMarkS-2+
Annotation Software rev.	6.3
Features Annotated:	Gene; CDS; rRNA; tRNA; ncRNA
Genes (total):	5,320
CDSs (total)	5,266
Genes (coding)	5,176
CDSs (with protein)	5,176
Genes (RNA):	54
rRNAs	2, 2, 2 (5S, 16S, 23S)
complete rRNAs	2, 2, 2 (5S, 16S, 23S)
tRNAs	44
ncRNAs	4
Pseudo Genes (total)	90
CDSs (without protein)	90
Pseudo Genes (amb. res.)	0 of 90
Pseudo Genes (frameshift)	25 of 90
Pseudo Genes (incomplete)	66 of 90
Pseudo Genes (internal stop)	10 of 90
Pseudo Genes (multiple probl.)	11 of 90
CRISPR Arrays:	6
